# A high latitude Devonian lungfish, from the Famennian of South Africa

**DOI:** 10.7717/peerj.8073

**Published:** 2019-12-04

**Authors:** Robert W. Gess, Alice M. Clement

**Affiliations:** 1 Geology Department, Rhodes University, Makhanda/Grahamstown, South Africa; 2 Albany Museum, Makhanda/Grahamstown, South Africa; 3 Wits University, DST-NRF Centre of Excellence in Palaeosciences (CoE-Pal), Johannesburg, South Africa; 4 College of Science and Engineering, Flinders University, Adelaide, SA, Australia

**Keywords:** Waterloo farm, Witpoort formation, Dipnoi, Famennian, South Africa, Western Gondwana, Sarcopterygii, Palaeozoic

## Abstract

New fossil lungfish remains comprising two parasphenoids, tooth plates and scales from the Famennian Witpoort Formation of South Africa are described. From the parasphenoid material, which bears similarity to *Oervigia* and *Sagenodus* but is nevertheless unique, a new genus, *Isityumzi mlomomde* gen. et sp. nov. is erected. Tooth plates and scales from the same locality may be conspecific but are not yet assigned until further material becomes available. The tooth plates closely resemble those of some taxa in the Carboniferous genus *Ctenodus*. The new taxon is significant as only the second Devonian lungfish described from the African continent, and for hailing from the high-latitude (polar) Waterloo Farm environment situated close to 70° south during the Famennian.

## Introduction

Lungfish (Dipnoi) are a clade of sarcopterygian (lobe-finned) fishes with their origins stretching back to the Early Devonian, over 410 million years ago (Campbell & Barwick, 1983; Chang & Yu, 1984; Denison, 1968; Wang et al., 1993). They reached a peak of diversity and abundance throughout the Devonian with close to 100 species described from that time period (Clack, Sharp & Long, 2011; Marshall, 1986a).

The vast majority of described Devonian taxa hail from Laurussia, with Gondwanan and China block taxa contributing just over one third of known species. Of those, eight are known from China (Qiao & Zhu, 2015), but the Gondwanan lungfish are dominated by Australian taxa (Long & Trinajstic, 2018) with over 25 described species, leaving just two remaining taxa; *Dipnotuberculus* from Mid-Devonian Moroccan deposits (Campbell et al., 2002) and *Iranorhynchus* from the Late Devonian Arabian Plate (Janvier & Martin, 1978), although the dipnoan affinities of the latter taxon have since been questioned (Marshall, 1986b). An unnamed lungfish palate from the Frasnian deposits in the Maider Basin north-west of Fezzou in Morocco is mentioned in Murray (2000) and we believe this most likely to be *Dipnotuberculus* (Campbell et al., 2002). However, it is unclear why the precise stratigraphic ages differ between reports.

Whereas all other Devonian lungfish bearing sites originated in tropical to warm temperate conditions, the Waterloo Farm lagerstätte, situated within the Famennian-aged Witpoort Formation (Witteberg Group, Cape Supergroup) (Gess, 2016), provides a unique window into a high palaeolatitude (i.e. polar) fauna, situated at approximately 70° latitude (Torsvik & Cocks, 2011; Torsvik & Cocks, 2013) or possibly even further south (Scotese & McKerrow, 1990). Sporadic systematic excavation and collection over three decades has produced an unusually complete record of a Famennian ecosystem, in part resulting from the presence of exceptional soft tissue preservation of both vegetative (Gess & Hiller, 1995a) and animal (Gess, Coates & Rubidge, 2006) origin.

The vertebrate fauna includes the only two known species of Devonian high-latitude tetrapods, *Tutusius* and *Umzantsia* (Gess & Ahlberg, 2018), the fossil lamprey *Priscomyzon riniensis* (Gess, Coates & Rubidge, 2006) and several placoderms, including an antiarch placoderm, *Bothriolepis africana*, phlyctaniid arthrodire placoderms *Groenlandaspis riniensis* (Long et al., 1997), *Africanaspis doryssa* (Gess & Trinajstic, 2017; Long et al., 1997) and *Africanaspis edmountaini* (Gess & Trinajstic, 2017). The fauna also includes a diplacanthid acanthodian *Diplacanthus acus* (Gess, 2001), a gyracanthid acanthodian (Gess & Hiller, 1995b), chondrichthyans *Plesioselachus acus* (Anderson et al., 1999; Gess & Coates, 2015b) and *Antarctilamna ultima* (Gess & Coates, 2015b), actinoperygians (Gess & Coates, 2008), the coelacanth *Serenichthys kowiensis* (Gess & Coates, 2015a) and a tristichopterid close to *Hyneria* (Gess & Coates, 2008).

Aquatic invertebrates are dominated by hundreds of valves of a fresh to brackish water mussel, *Naiadites* form Devonica (Scholtz & Gess, 2017). Interpretation of the depositional environment as a back barrier coastal lagoonal estuary with fresh and marine influences (Gess & Hiller, 1995b) is supported by the abundant presence of both marine-type phaeophyte algae (Hiller & Gess, 1996) and fresh to brackish water indicative charophyte algae (Gess & Hiller, 1995a).

Despite its high latitude setting the climate does not seem to have been overly extreme; a complex adjacent terrestrial habitat included the progymnosperm tree, *Archaeopteis notosaria* (Anderson, Hiller & Gess, 1994, 1995) as well as rhizomorphic lycopods such as the widespread *Leptophloem rhombicum* (Gess & Hiller, 1995b; Prestianni & Gess, 2014) and the endemic *Kowieria alveoformis* (Gess & Prestianni, 2018). The terrestrial environment also supported invertebrate life such as the scorpion *Gondwanascorpio umzantsiensis*, the oldest known terrestrial animal from Gondwana (Gess, 2013).

Although not abundantly represented, the lungfish material we present below contributes to a better understanding of this unique high-latitude environment and provides the only record of Late Devonian lungfish remains from western Gondwana (South America and Africa).

## Materials and Methods

All the material was collected from a single black metashale lens (the MFL or Main Fish Layer) at Waterloo Farm to the south of Makhanda/Grahamstown, Eastern Cape, South Africa, situated at 33°19′24.24″S, 26°32′13.39″E. This deposit is located within the upper portion of the predominantly arenaceous Witpoort Formation (Witteberg Group, Cape Supergroup), a formation corresponding to the Famennian stage of the Late Devonian (Gess, 2016). The black metashale is interpreted as a derivative of muds deposited in a back barrier estuarine environment (Gess & Hiller, 1995a).

Specimens were collected by Dr. Robert Gess between 1999 and 2017 from a large sample of shale rescued from roadworks during 1999, with the exception of AM7530 excavated in 2015 by Mr. Chris Harris from shale rescued by Dr. Gess in 1999 and AM4821 collected by Mrs. Sheila Coutouvides in 1989 (Gess & Hiller, 1995b).

All specimens are represented by near two-dimensional compressions preserved within black carbonaceous metashale. All organic material was replaced by secondary metamorphic mica during diagenesis, which has largely been replaced by kaolin and/or chlorite with uplift.

The described material comprises four blocks of part and counterpart, and one single block. The lungfish specimens are represented by a complete parasphenoid, a partial parasphenoid, two tooth plates associated with prearticulars and numerous scales. Specimens were studied using a binocular microscope and photographed using a Nikon D7500 with a Nikon 60 mm macro lens. Labelled line drawings were made both directly from the specimens and from photographs.

### Results: systematic palaeontology

OSTEICHTHYES Huxley, 1880

SARCOPTERYGII Romer, 1955

DIPNOMORPHA Ahlberg, 1991

DIPNOI Müller, 1844

*Isityumzi mlomomde* gen. et sp. nov.

*Diagnosis*. Lungfish with parasphenoid corpus and stalk equal in length, stalk narrow with parallel sides tapering to a single point. Corpus almost as wide as long and with a broad anterior angle of 90°.

*Etymology/Derivation of name*. Generic name ‘*Isityumzi’*, from isiXhosa language meaning a device for crushing (from ukutyumza, to crush). Specific name ‘*mlomomde’* from isiXhosa meaning ‘long mouthed’.

*Holotype*. Complete parasphenoid, AM6501, Albany Museum, Grahamstown/Makhanda, Eastern Cape, South Africa.

*Other material*. AM 4821 (partial parasphenoid).

*Horizon and type locality*. Waterloo Farm, Grahamstown/Makhanda, South Africa; Witpoort Formation, Witteberg Group, Famennian, Late Devonian.

*Note*. The new taxon is erected from the parasphenoid material alone. Although we expect that the tooth plate and scale material are conspecific, we do not formally assign them to *Isityumzi mlomomde* gen. et sp. nov. here.

The electronic version of this article in Portable Document Format will represent a published work according to the International Commission on Zoological Nomenclature (ICZN) and hence the new names contained in the electronic version are effectively published under that Code from the electronic edition alone. This published work and the nomenclatural acts it contains have been registered in ZooBank, the online registration system for the ICZN. The ZooBank Life Science Identifiers can be resolved and the associated information viewed through any standard web browser by appending the LSID to the prefix http://zoobank.org/. The LSID for this publication is: urn:lsid:zoobank.org:pub:0D8B5681-C4F6-43E3-A7E0-F2F9199AD1FC. The LSID for *Isityumzi* gen. nov. is urn:lsid:zoobank.org:act:A2B074C1-12C3-4AE3-9F7F-26FA6B4DCBE7 and that for *mlomomde* sp. nov. is urn:lsid:zoobank.org:act:9B17F871-21DB-4B64-9B72-EF54EF4934DF. The online version of this work is archived and available from the following digital repositories: PeerJ, PubMed Central and CLOCKSS.

### Results: description

*Parasphenoid*. Two parasphenoids of *Isityumzi mlomomde* gen. et sp. nov. are preserved, the larger of which is incomplete and has previously been published (Anderson, Hiller & Gess, 1994; Gess & Hiller, 1995b). The description herein instead relies more heavily on the complete second specimen. The parasphenoid is a thin, flat element, not fused with the pterygoids but showing a clear area for overlap by these bones. There is a considerable size difference between the two specimens, with AM 4821 measuring 36 mm across the widest point and AM 6501 just 20 mm. The anterior section is wide (almost half the entire length) and the anterior angle is broad at 90°. The parasphenoid has a distinct broad rhombic anterior portion (corpus) and a narrow posterior shaft (stalk). The corpus and stalk are equilateral in length. The stalk is slender, has parallel sides and tapers to a single point posteriorly. The lateral angle between the stalk and the corpus is 30°. There is a long median ridge running anteroposteriorly, which carries a broad channel running the length of the stalk, although it is unclear if this lies on the dorsal or ventral surface. There is no dental material on the parasphenoid, nor is there any obvious indication of a buccohypophysial opening.

**Figure 1:**
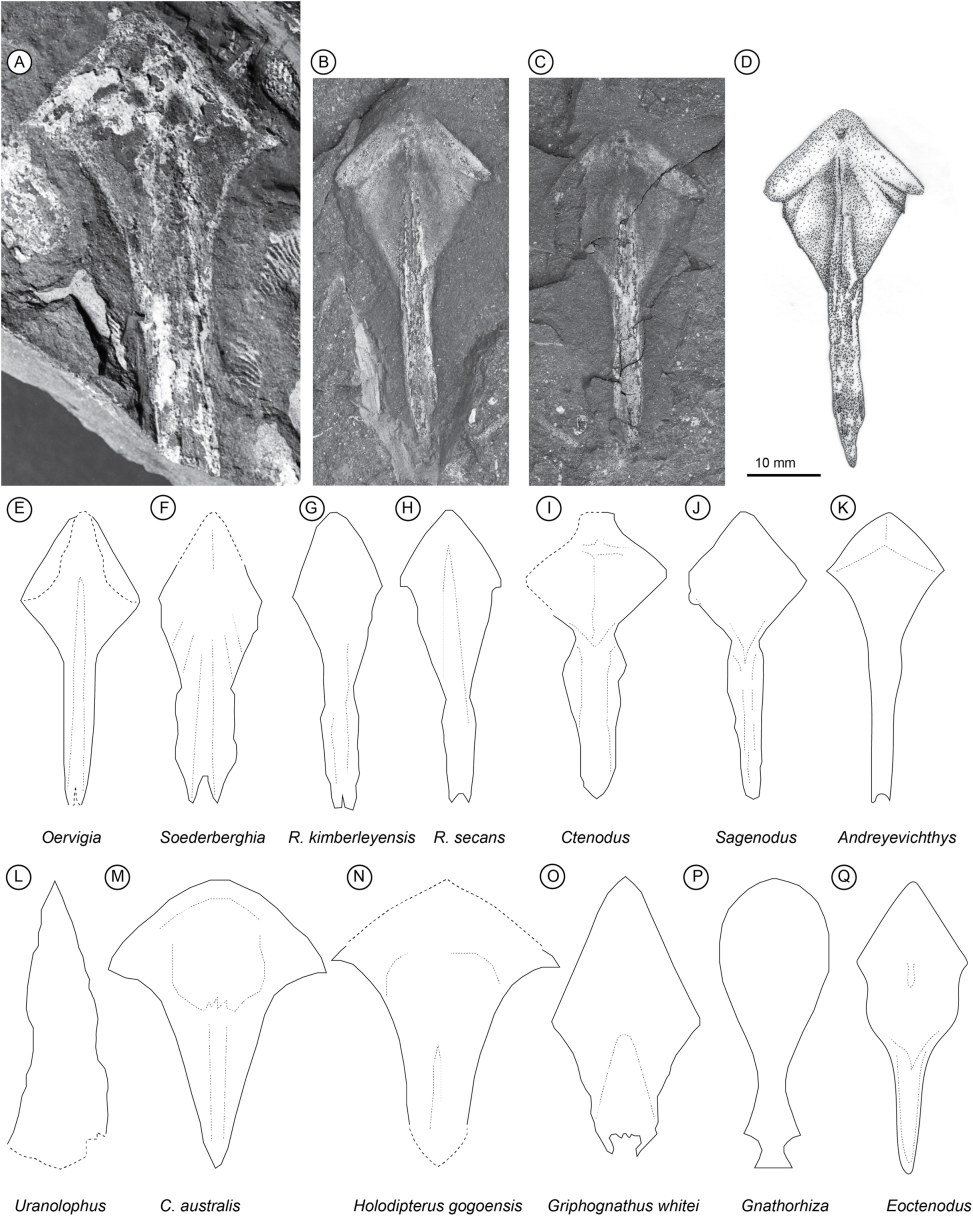
Palaeozoic Lungfish Parasphenoid Morphology. *Isityumzi mlomomde* n. gen. et sp., (A) partial parasphenoid (AM 4821); (B) and (C) part and counterpart of the holotype (AM 6501a/b); (D) interpretive drawing of the holotype. Comparative parasphenoid outlines: (E) *Oervigia nordica* (Lehman, 1959, Fig. 27), (F) *Soederberghia groenlandica* (Lehman, 1959, Fig. 17), (G) *Rhinodipterus kimberleyensis* (Clement, 2012, Fig. 3), (H) *Rhinodipterus secans* (Gross, 1956, Fig. 20), (I) *Ctenodus cristatus* (Sharp & Clack, 2013, Fig. 16), (J) *Sagenodus copeanus* (Schultze & Chorn, 1997, Fig. 22), (K) *Andreyevichthys epitomus* (Krupina, 1987, Fig. 2), (L) *Uranolophus* (Denison, 1968, Fig. 8), (M) *Chirodipterus australis* (Miles, 1977, Fig. 76), (N) *Holodipterus gogoensis* (Miles, 1977, Fig. 77), (O) *Griphognathus whitei* (Miles, 1977, Fig. 75), (P) *Gnathorhiza* sp. (Berman, 1976, Fig. 4), and (Q) *Eoctenodus microsoma* (Long, 1987, Fig. 3). Parasphenoids not drawn to scale, redrawn from references given within.

*Parasphenoid: remarks*. In overall proportions the parasphenoid of *Isityumzi* most closely resembles that of *Oervigia* (Lehman, 1959), although it lacks the bifid stalk of that taxon (Fig. 1). The stalk tapers into a single point posteriorly like *Sagenodus* (Schultze & Chorn, 1997) or *Ctenodus* (Sharp & Clack, 2013). The stalk is slender as in *Oervigia* (Lehman, 1959), but not quite as elongate as *Andreyevichthys* (Krupina, 1987, Fig. 2) and *Sagenodus* (Schultze & Chorn, 1997), being equal in length with the corpus. The stalk lacks lateral expansions seen in *Soederberghia* (Lehman, 1959), *Ctenodus* (Sharp & Clack, 2013) and *Orlovichthys* (Krupina, Reisz & Scott, 2001, Fig. 2). Unlike some other long-headed lungfish (*Griphognathus*, Miles, 1977, Fig. 75; *Jarvikia* and *Soederberghia*, Lehman, 1959, Figs. 24 and 29), the parasphenoid has a highly distinct rhombic corpus (‘lozenge’), which is sharply differentiated from a narrow posterior stalk. *Isityumzi* lacks an obvious buccohypophyseal opening as is found in older lungfish such as *Dipterus* (Jarvik, 1980, Fig. 307) and *Dipnorhynchus* (Campbell & Barwick, 2000, Fig. 1). However, a darkened area near the anterior angle of the parasphenoid may represent the ventral-most part of the hypophysial canal, like that in ‘*Chirodipterus’ australis* (Miles, 1977).

### Results: other lungfish material

*Other material*. AM 5863a/b (prearticular and partial tooth plate); AM7530a/b (prearticular and tooth plate); and AM 7531a/b, AM 7532 a/b, AM 7533 a/b, AM 7534, AM 7535, AM 7536, AM 7537 a/b/c (isolated scales).

*Horizon and type locality*. Waterloo Farm, Grahamstown/Makhanda, South Africa; Witpoort Formation, Witteberg Group, Famennian, Late Devonian.

**Figure 2:**
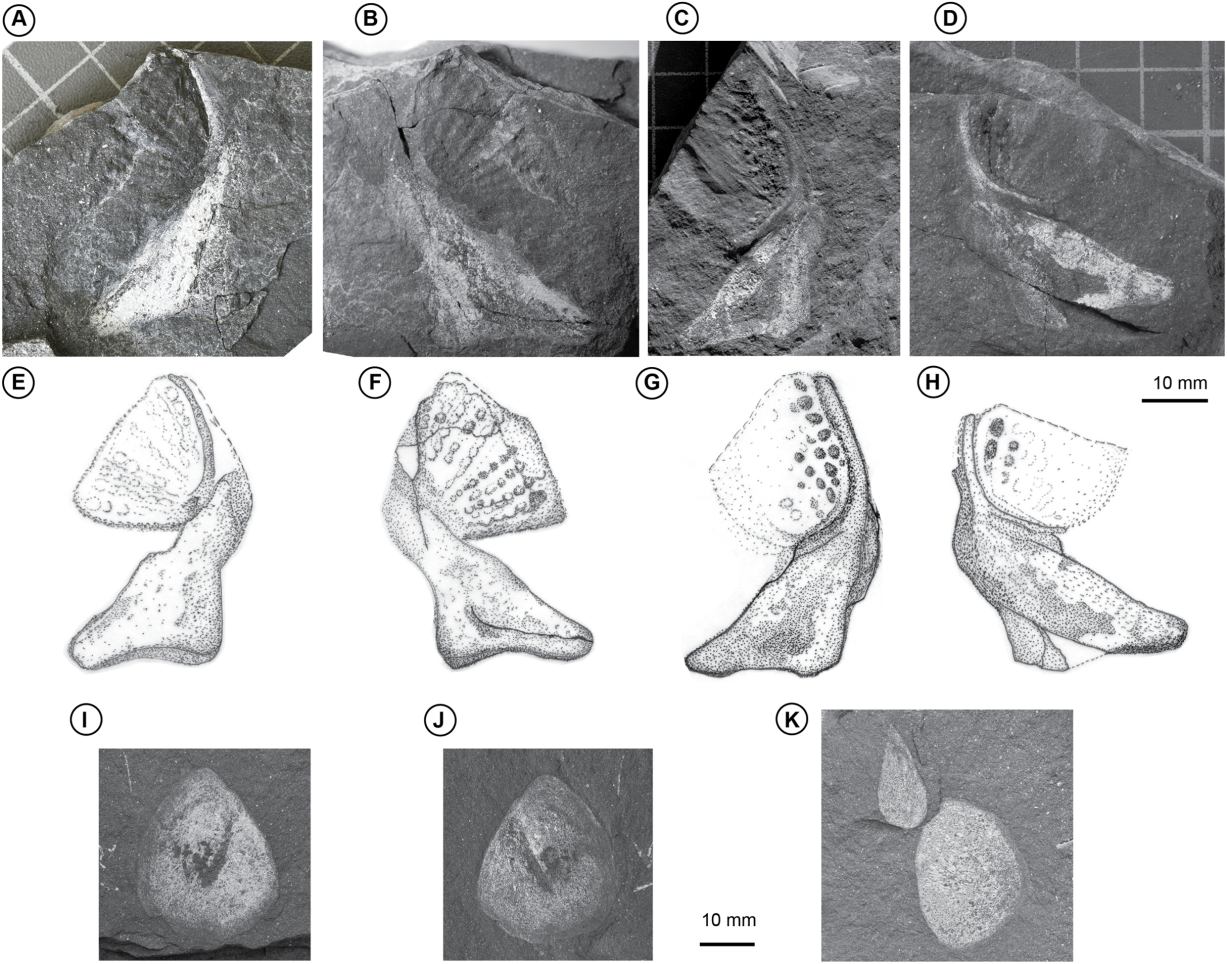
Other lungfish material. (A) and (B) toothplates, (AM7530a/b); toothplates, (C) and (D) (AM 5863a/b); (E–H), interpretive drawings of (A–D); photographs of scales shown in (I–J) (AM 7531a/b) and (K) (AM 7537a). Scale bars all 10 mm.

*Tooth plates*. There are two tooth plates preserved, both with part and counterpart (Fig. 2). We do not herein formally assign them to *Isityumzi mlomomde* gen. et sp. nov. until further material becomes available to enable unambiguous identification. They are of the type containing radiating tooth rows with separate cusps and with part of the supporting bone attached. It is difficult to discern how many tooth rows were present on AM 5863, but WF-MFL preserves this detail better and shows at least eight (but possibly nine) tooth rows with at least six low cusps per row. The lateral-most cusp tends to be larger and isolated from the remainder of the ridge. The tooth rows curve posteriorly so that they diverge from a common point; the outermost rows curving the most to form the fan-shaped tooth plate. The angle between the first and last rows is 65°. Both tooth plates are attached to the supporting prearticular bone, although a groove (lingual furrow) is visible between them. It is not clear if the symphysial surface for contact with the opposing prearticular was broad or narrow. The prearticular bears straight mesial and posterior edges to form an obtuse triangle in outline. The cavity for the Meckelian bone may be represented by a darker patch central within the flange on AM 5863. The apparent outline of AM7530 measures ∼26 mm by ∼16 mm.

*Tooth plates: remarks*. The preservation of material from Waterloo Farm is unusual. Across all vertebrate taxa dentine is not as well preserved as bone (which is itself often flattened) though, by contrast, cartilage is often well preserved. Impressions of the tooth plate*s* do not therefore allow discernment as to whether or not the cusps were sharply-pointed as in *Orlovichthys* (Krupina, Reisz & Scott, 2001) and *Oervigia* (Lehman, 1959, Fig. 17A), nor whether they were strongly convex (cf. Smithson, Richards & Clack, 2015). The new tooth plates do not possess long anteromedial dentine extensions to the tooth plate like those in *Orlovichthys* (Krupina, Reisz & Scott, 2001), *Rhinodipterus* (Clement, 2012) and *Andreyevichthys* (Krupina, 1987). There is no evidence of a prominent ascending process on the prearticular, in contrast to *Sinodipterus* (Qiao & Zhu, 2009) although, as the material is flattened we can’t unequivocally rule this out. The tooth plates are not triangular in outline as is common in numerous Devonian taxa (e.g. *Dipterus*, White, 1965; *Harajicadipterus*, Clement, 2009; *Sinodipterus*, Qiao & Zhu, 2009; *Adelargo*, Johanson & Ritchie, 2000), nor ovoid (e.g. *Eoctenodus*, Long, 1987), but instead they are fan-shaped, more reminiscent of *Ctenodus cristatus* (Sharp & Clack, 2013, Fig. 1C), although possessing one less tooth row than that species. In fact the emended generic diagnosis for *Ctenodus* based as it is entirely on tooth plate morphology (Sharp & Clack, 2013), could apply to the tooth plates of this material. However, due to the difference between the parasphenoid of *Isityumzi* and those known from *Ctenodus* species, we have been cautious in not assigning the material to *Ctenodus* and thereby potentially erroneously distorting its temporal and biogeographic range. The similarity of the tooth plates to those of *Ctenodus* does however lend further support to the hypothesis that the Devonian-Carboniferous boundary was less rigid for lungfish than previously understood (Clack et al., 2018).

*Squamation*. Some thin, smooth cycloid lungfish body scales are also preserved (Fig. 2). The scales are sub-rounded tending towards subtriangular and appear to have had an ornament of small tuberosities/denticles. One of the largest examples, AM 7532, measures 50 mm across the major axis and 36 mm across minor axis. Due to the nature of preservation, it is unclear whether cosmine was present or not.

## Discussion

Seemingly it wasn’t until the Mesozoic that lungfish began to diversify in Africa (e.g. *Ceratodus* spp., *Ptychoceratodus, Mioceratodus, Asiatoceratodus*) and by the Cretaceous Period they were widespread (Longrich, 2017; Murray, 2000). Indeed, one of the three living genera of lungfish (*Protopterus*) is thought to have existed in Africa for at least 100 million years (Otero, 2011). However, their Palaeozoic history has a depauperate record in Africa. Aside from the new taxon and material described herein, the only other undisputed Devonian lungfish from the continent is *Dipnotuberculus*, hailing from Morocco some ∼10,000 km away (Campbell et al., 2002). At this time Morocco would have been situated much closer to the equator in the temperate/sub-tropical zone (with a latitude between 30 and 60 degrees), whereas South Africa had a very high latitude location close to the South Pole (Scotese & McKerrow, 1990; Torsvik & Cocks, 2013).

The vast majority of described Devonian lungfish fossils are from subtropical and tropical locations much closer to the equator (including all of those from Australia, Laurussia and China). Tropical locations, in particular reefs, have long been recognised as centres of maximum diversity throughout geological history (Renema et al., 2008). Conversely, taxa living in high-latitude ‘polar’ regions (e.g. >60° in latitude) are in fact specifically investigated to understand the explicit physiological adaptations that enable them to survive in those extreme environments (Woodward, Rich & Vickers-Rich, 2018). Thus, quite significantly *Isityumzi* represents the first ever high-latitude lungfish known. Today, equivalent polar zones experience very low mean annual temperatures and months of extended winter darkness, which might contribute to the lack of lungfish at these sites.

*Isityumzi* joins several other Devonian genera in sharing a long skull morphology (e.g. *Rhinodipterus, Andreyevichthys, Oervigia, Orlovichthys, Iowadipterus*). In fact, possession of an elongate skull appears to completely dominate Upper Devonian lungfish morphologies across the globe, and particularly so during the Famennian (with perhaps *Apatorhynchus* the exception, see Friedman & Daeschler, 2006); short-snouted taxa such as the ‘chirodipterids’ had all but disappeared by this time.

It has long been noted that lungfish and tetrapods co-occur in Late Devonian deposits (Friedman & Daeschler, 2006; Lebedev, 2004; Thomson, 1980), most frequently in continental ecosystems. Of the 15 named Devonian tetrapod genera (Gess & Ahlberg, 2018; Olive et al., 2016), only four (*Jakubsonia, Obruchevichthys, Sinostega* and *Weberepeton*) do not co-occur with *Soederberghia* or some other long-headed lungfish (e.g. *Andreyevichthys, Orlovichthys*). In fact, it in only *Jakubsonia* that reportedly occurs with lungfish (*Holodipterus, Dipterus, Conchodus*), but not one of the long-headed forms.

Sharp & Clack (2013) suggested that lengthened skull roof E-bones in the Carboniferous lungfish *Ctenodus* could be related to increased strength of the rostral area to support the palate for a durophagous lifestyle. Perhaps it was the universal fixation of this feeding strategy that explains the widespread ‘long-headed, tooth-plated’ lungfish morphotype present during the Famennian. However, the short-snouted ‘chirodipterids’ common in the Frasnian possessed large, flat hypermineralised tooth plates and crania well-suited to high mechanical advantage likely even better suited to durophagy. This raises the possibility that the adoption of the common long-headed morphotype might have been related to another behaviour, possibly aerial respiration, as has been previously postulated (Ahlberg, Johanson & Daeschler, 2001; Campbell & Barwick, 1988; Clement, 2012; Clement & Long, 2010; Clement et al., 2016; Long, 1993; Thomson, 1971). The spread of long-headed lungfish in time and space closely associated with tetrapods that were also developing their own adaptations related to aerial respiration is likely salient and warrants further investigation.

Furthermore, it is notable that whereas most comparable environments contain abundant lungfish remains, these are extremely uncommon at Waterloo Farm. Out of approximately 400 vertebrate specimens (excluding scales) only four are attributable to lungfish. Considering the abundance of lungfish in contemporaneous Devonian deposits, and in the presence of hundreds of mussel shells this seems surprising.

A possible explanation is suggested by the exceptionally large size of the phlyctaeniid arthrodire *Groenlandaspis riniensis*, the largest species attributed to this genus (Long et al., 1997). For example, a large isolated anterior lateral plate of this species (AM6582) measures 150 mm along its spinal plate contact, suggesting a total length of head and trunk armour of 480 mm according to the proportions reconstructed by Long et al. (1997). This suggests a large fish with a total body length of approximately 1 m. The dentition of this species consists of supragnathals and infragnathals consistent with a durophagous lifestyle. It is therefore possible that *Groenlandaspis riniensis*, which represents the most commonly preserved vertebrate taxon at Waterloo Farm, inhibited abundance of other durophagous species, namely lungfishes.

## Conclusions

- *Isityumzi* represents the only record of Late Devonian lungfish remains from western Gondwana (South America and Africa) and is described from its parasphenoid.
- The parasphenoid of *Isityumzi* bears similarity to *Oervigia* and *Sagenodus* but differs from those taxa in having a single pointed stalk (c.f. *Oervigia*) and the corpus and stalk being equal in length (c.f. *Sagenodus*).
- The lungfish tooth plates from the same horizon and locality are similar to the Carboniferous taxon *Ctenodus cristatus*.
- The new genus is the first Devonian lungfish described from a high-latitude environment, and the only lungfish known from the Witpoort Formation.
- It appears likely that *Isityumzi* conforms to a common morphotype for Famennian lungfishes in possessing a long head and tooth plates bearing radiating ridges.
- *Isityumi* adds further evidence for the co-occurrence of lungfish with tetrapod taxa.
- It is possible that niche competition with the abundant and unusually large resident *Groenlandaspis* may account for the uncharacteristically low abundance of lungfish remains.

## Additional Information and Declarations

### Data Availability

The following information was supplied regarding data availability: All photographs of the specimens are available at CLEMENT, Alice; Gess, Rob (2019): Specimen PHOTOS. figshare. Figure. https://doi.org/10.6084/m9.figshare.9162227.v1. All fossil material is deposited in public collections at the Albany Museum, Grahamstown, Eastern Cape, South Africa. The Holotype is desinated as AM6501, complete parasphenoid. Other material. The specimens include AM 4821 (partial parasphenoid); AM 5863a/b (prearticular and partial tooth plate); AM 6500a/b (partial skull roof); AM 6501a/b (complete parasphenoid); AM7530a/b (prearticular and tooth plate); and AM 7531a/b, AM 7532 a/b, AM 7533 a/b, AM 7534, AM 7535, AM 7536, AM 7537 a/b/c (isolated scales).

### New Species Registration

The following information was supplied regarding the registration of a newly described species: Publication LSID: urn:lsid:zoobank.org:pub:0D8B5681-C4F6-43E3-A7E0-F2F9199AD1FC. Isityumzi gen. nov. LSID: urn:lsid:zoobank.org:act:A2B074C1-12C3-4AE3-9F7F-26FA6B4DCBE7. mlomomde sp. nov. LSID: urn:lsid:zoobank.org:act:9B17F871-21DB-4B64-9B72-EF54EF4934DF.
